# Characterization of the Potential Long-Term Impact from Sedimentary PFAS at a Historically Contaminated Textile Waste Site

**DOI:** 10.1021/acsestwater.5c01210

**Published:** 2025-12-29

**Authors:** Jarod Snook, Jitka Becanova, Simon Vojta, Rainer Lohmann

**Affiliations:** Graduate School of Oceanography, 54083 University of Rhode Island, 215 S Ferry Road, Narragansett, Rhode Island 02882, United States

**Keywords:** PFAS, sediment, mass flux, environment, human health

## Abstract

Per- and polyfluoroalkyl substances (PFAS) are pervasive pollutants at historically contaminated sites throughout the United States and beyond. Two such sites in Rhode Island, USA, are textile-mill-associated waste retention ponds known to introduce PFAS contamination to the adjacent river, estuary, and eventually the Atlantic Ocean. Here, we thoroughly investigated the retention ponds as a long-term source of PFAS via water passive sampling, sediment coring, and laboratory-derived partitioning coefficients, *K*_d_, with field sediment and water. Additional studies were performed to assess the mobility and estimate the mass fluxes of PFAS from sediment to water. Retention pond 1 was more contaminated (up to 26 ng/L PFOA in water and 74 ng/g PFTrDA in sediment). Derived log *K*_d_ values ranged from 1 to 5 for most PFAS, indicating a shift from relative mobility to high storage potential in sediment. Estimated loss fluxes from the sediment varied between 5 and 228 μg m^–2^ year^–1^, resulting in desorption times from 3 years for FPeSA to >100 years for FOSA. The combined evidence suggests that this textile mill retention pond, if left untreated, constitutes a source of long-term contamination to the river.

## Graphical abstract

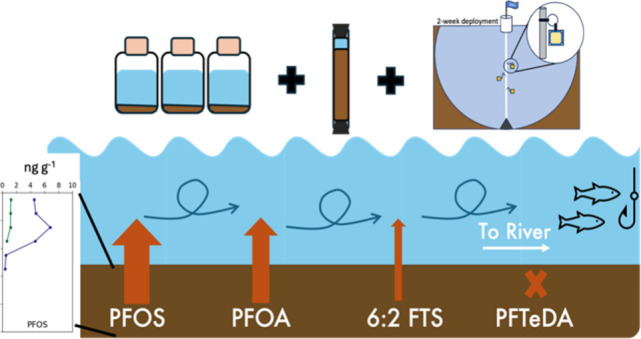

## Introduction

Per- and polyfluoroalkyl substance (PFAS) pollution, a well-established ecological and human health threat, is widespread in U.S. natural waters.^1–3^ PFAS have been detected in rivers, lakes, groundwater, estuaries, and coastal oceans in all regions of the United States over the last two decades.^4–7^ PFAS contamination of natural water may originate from several widely varied sources, e.g., aqueous film-forming foam application, industrial manufacturing, wastewater, and/or landfill leachate.^8,9^ Considering the many sources of PFAS, usually with differing source “fingerprints”, it is difficult to identify and manage PFAS pollution effectively.^10–12^ For already contaminated sites, concentrations exceeding certain thresholds (depending on location/enforcement bodies) may be subject to expensive and invasive remediation.^13–15^

Despite phase outs of select PFAS, historically contaminated sites can be sources of ongoing pollution.^16–18^ As PFAS are long-lived and sorb to sediments, the storage and eventual leaching of contaminated sediments can impair ecological and human health for decades. PFAS sources hydrologically connected to larger water bodies can transport contamination over time.^19–21^ An example of this is the Pawcatuck River in Rhode Island, USA,^12,22^ where a long-term study identified two sampling sites that were particularly impacted by PFAS—the sources for each were suspected to be industrial textile mill retention ponds along, then eventually joining, the river. There are no wastewater treatment facilities^23^ or other major sources of PFAS discharge to the upstream Pawcatuck River; thus, the textile mill lagoons are potentially the most important sources for the majority of the river’s length.

To better understand these sites and demonstrate an effective method for characterizing long-term contamination potential, we conducted several investigations to thoroughly characterize the PFAS content and interactions in each. This process, while site-specific, demonstrates a simple conceptual process to evaluate lasting contamination from many other such sites nationally and globally. Specifically, our aims were to (1) determine surface water and sediment PFAS concentrations, (2) characterize the PFAS’ sediment-water partitioning *K*_d_,^24,25^ and (3) assess the potential and time-scales of PFAS to be remobilized from the sediment via diffusion.

## Methods

### Field Sites

Two field sites (see Supplemental Map and Supplemental Text 1) were selected based on the previous findings in Dunn et al. Retention pond 1 (RP1) in Bradford, Rhode Island is one in a series of ponds used as settling lagoons for a nearby textile mill between the 1960s and the 1980s.^26^ Retention pond 2 (RP2) in Westerly, Rhode Island is a retention pond/canal with a separate PFAS signature and currently active nearby textile mill.^27^

### Water Column Deployments

Dissolved PFAS concentrations were measured via a diffusive gradient in thin-film (DGT) passive sampling.^28^ In short, agarose-based samplers were placed in the center of the pond for a total of 14 days on rope lines held vertically by a weight and float system. Duplicate DGTs were placed at each of the top, bottom (near sediment), and middle of the water column (six samplers were deployed at each site). A total of three field blanks were used over both sites. Extraction details for various sample types are included in Supplemental Text 2. Sampling rates^28^ (mL day^–1^, Table S1) were used to convert the mass detected on the sampler (*M*_s_, ng) to time-weighted-average dissolved PFAS concentration (*C*_w_, ng mL^–1^) via eq 1.

$$C_{w}=\frac{M_{s}}{R_{s}\times t}$$ 1

### Sediment Coring and Characterization

Sediment samples were obtained via hand sediment coring, which provides depth-integrated PFAS concentration information via analysis of individual sections. Two-inch diameter polypropylene sediment corers were pushed into sediment to a depth of 15–20 cm, then capped, and removed to obtain an intact core (one per site). Cores were sectioned at 1 cm intervals where fine sediment was present and then 2–3 cm intervals when the substrate transitioned to inorganic sand. Sections were freeze-dried and then sieved though a 1 mm sieve to remove large debris and rocks and between 0.8 and 1.8 g extracted for PFAS (Supplemental Text 2).

For sections where PFAS were detected (above MDLs), additional freeze-dried sediment (9–20 mg) was sieved in a 1 mm sieve, acidified with sulfurous acid to remove any inorganic carbon, and analyzed for organic carbon (OC) content with a Costech ECS 4010.

### Laboratory *K*_d_ Experiment

Using additional sediment and water grab samples, duplicate *K*_d_ determination experiments were conducted for RP1.^24^ In HDPE bottles, 2 g (d.w.) of freeze-dried sediment was combined with 500 mL of water from the field (no PFAS were spiked) and shaken (100 rpm) for 7 days. Water and sediment phases were separated by filtration and extracted separately (Supplemental Text 2). After quantification, compound-specific *K*_d_ (mL g^–1^) values were calculated via eq 2, with *C*_s_ being the sediment PFAS concentration (ng g^–1^) and *C*_w_ being the water PFAS concentration (ng mL^–1^).

$$K_{d}=\frac{C_{s}}{C_{w}}$$ 2

### LC-MS/MS Analysis and QA/QC

Concentrated methanol extracts from all sample types were diluted by a factor of 2.5 into a final 100 μL solution of 40:60 LC-MS methanol:10 mM ammonium acetate in LC water. These were analyzed via LC-MS/MS (SCIEX ExionLC AC UHPLC coupled to a SCIEX X500R quadrupole time-of-flight tandem mass spectrometer) and quantified via isotope dilution of extracted internal standards (EIS, similar to the process in EPA Method 1633). SCIEX data processing software was used to quantify the results (more details on LC-MS/MS operation are available in Supplemental Text 3, and the full target compound list is in Table S2).

Method detection limits (MDL, Table S3) for field samples were calculated via the average +3 × the standard deviation of process blank samples with detected PFAS for each sample type. For PFAS with no detections in blank samples, the instrumental detection limit (signal-to-noise ratio = 3) was used as the MDL. Samples below MDL thresholds were omitted or reported as “<MDL” as appropriate. All blanks were below MDL values for each sample type. Due to some PFAS detections in the *K*_d_ experiment process blank, MDL values for the *K*_d_ experiment were set to be 10× the process blank detection amount, or the instrumental detection limit, as applicable. As nonextracted internal standards (NIS) were also added prior to analysis, EIS recovery values for field samples were calculated via comparison of EIS and NIS area ratio to calibration curve samples (similar to EPA Method 1633 ^29^). Average EIS recoveries were within 64–173% for target PFAS with DGT and 73–200% for sediment core sections (Table S4, EIS recovery values are mapped to corresponding native PFAS for each PFAS detection).

### Modeling Approach

The modeling approach combined several concepts from Schwarzenbach et al. to ultimately obtain an estimate of PFAS mass flux from sediment (see Figure 1 for a visualization of model inputs and processes). For sediment to act as a potential source of PFAS back to the water column, the PFAS travel distance must exceed the sedimentation rate of the retention pond for PFAS export from sediment to the overlying water to occur. The half-concentration penetration distances of a hypothetical two-layer sediment system (one “clean” and one containing constant PFAS concentration) were calculated via^30^

$$x_{1/2}=\sqrt{\frac{D_{w}t}{1+r^{\mathrm{sc}}K_{d}}}$$ 3

where *r* ^sc^ is the solid to water phase ratio (kg L^–1^), *K*_d_ is the distribution coefficient (L kg^1–^), *D*_w_ is the water diffusion coefficient (cm^2^ s^–1^), and *t* is the time (s). *D*_w_ was calculated as the average of two models:^30^

$$D_{w}=0.000152\times V^{-0.64}$$ 4

$$D_{w}=0.00007\times M^{-0.45}$$ 5

where *M* is a PFAS’ molecular weight and *V* is its molar volume.

**Figure 1:**
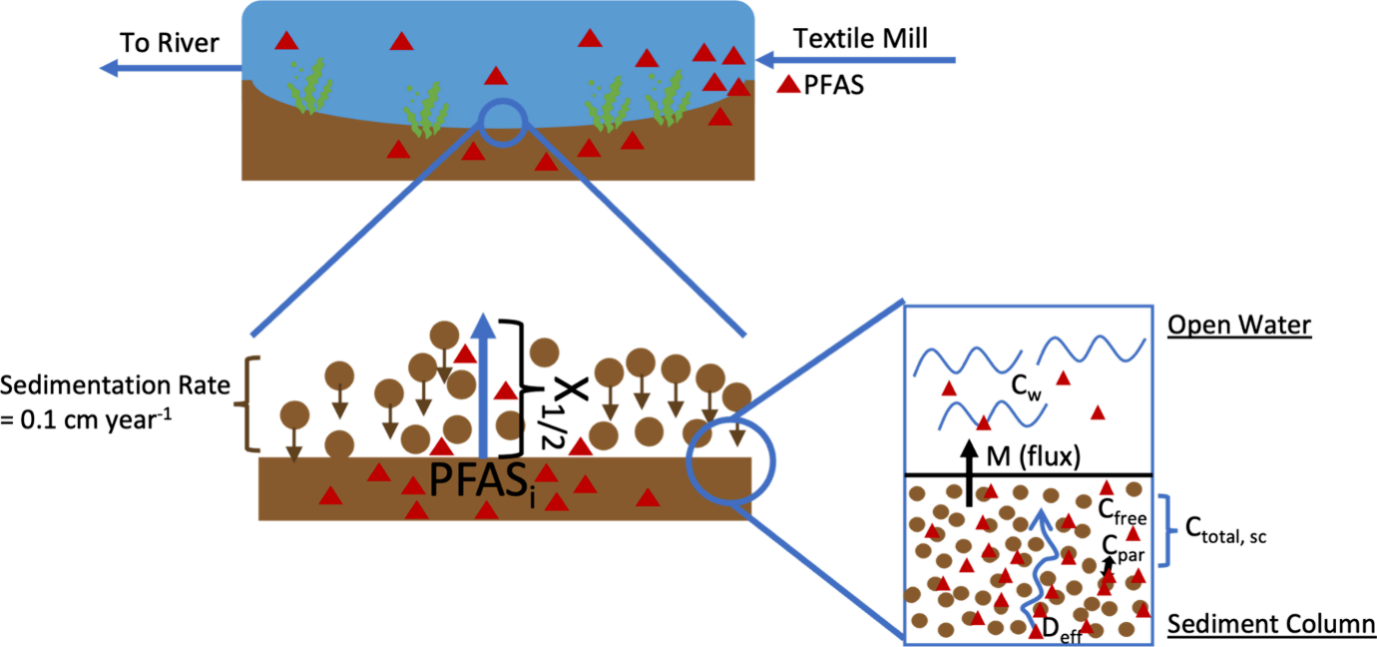
System and model diagram including a comparison of sedimentation rate vs PFAS diffusion travel distance (*x*_1/2_), as well as mass flux (*M*) from sediment to water based on various inputs. Red symbols represent a given PFAS.

Mass flux from sediment to water at RP1 was calculated via a series of models describing a two-box simulation with a wall boundary, the sediment column portion being a mixed-phase between sediment and water, as described in by Schwarzenbach et al.^30^ Effective diffusion (similarly used in the PFAS transport distance calculation) considering pore space and retardation by partitioning is given by eq 6.

$$D_{\mathrm{eff}}=\frac{D_{w}}{1+r^{\mathrm{sw}}K_{d}}$$ 6

with *D*_w_ being the diffusion coefficient in pure water (estimated by two empirical models), *r* ^sw^ being the solid to liquid phase ratio (via porosity and density, 0.67 and 2.63 kg L^–1^, respectively, measured by centrifuging field samples), and *K*_d_ being the partition coefficient of the given PFAS.

The overall quantity for mass flux was then calculated as

$$M=1.13(C_{\mathrm{eq},\mathrm{total}}^{\mathrm{sc}}-C_{\mathrm{total}}^{\mathrm{sc}})\times \sqrt{D_{\mathrm{eff}}t}$$ 7

with *C* ^sc^_eq,total_ being the total concentration of a given PFAS held in the sediment column (sediment and porewater) at equilibrium with respect to the measured surface water concentration (see below) and *C* ^sc^_total_ being the actual concentration of PFAS measured at 0–1 cm of the sediment and porewater (the porewater amount was determined by porosity and assumed to be in equilibrium with surrounding sediment). A positive flux indicates flux into the sediment; thus, the signs are reversed in the results for clarity. *C* ^sc^_eq,total_ was calculated by eq 8.

$$C_{\mathrm{eq},\mathrm{total}}^{\mathrm{sc}}=C_{w}\varphi \times (1+r^{\mathrm{sw}}K_{d})$$ 8

with *C*_w_ being the measured surface water concentration (ng/mL) and φ being the porosity (%).

A sensitivity analysis was conducted to better understand the extent that each input’s uncertainty affects the travel distance and flux model calculations. The impact of changing the parameters *K*_d_, *D*_w_, *r* ^sw^, measured *C*_w_ and *C*_s_, and φ by ±30% error was tested for each variable individually. For each PFAS, the model was rerun with 30% increased and decreased *K*_d_ values, and the change in flux output was quantified as the percent increase from the original model results. This was repeated for each individual variable and both model outputs to generate tables of relative model sensitivities for each (Table S5).

## Results and Discussion

### PFAS in the Water Column

Four passive samplers were recovered at RP1 from three depths, and two samplers were recovered from two depths at RP2. No differences in measured PFAS concentration were found between sampling depths (all values were within the average ±2 × standard deviation for individual PFAS and ΣPFAS). Well-mixed PFAS concentrations in the water are reasonable considering the shallow depth of the pond. Thus, water concentrations from all depths were averaged for each site (Table 1).

**Table 1:** Average and Standard Deviation of PFAS Concentrations at Each Site as Measured by DGT Passive Sampling^a^

compound	RP1_(*n*)_ (ng L^–1^)	RP1 St. Dev.	RP2_(*n*)_ (ng L^–1^)	RP2 St. Dev.
PFPrA*	0.33_(4)_	0.14	2.73_(1)_	NA
PFBA	2.64_(4)_	0.19	0.73_(2)_	0.31
PFPeA	5.02_(4)_	0.52	1.03_(2)_	0.36
PFHxA	7.36_(4)_	1.10	1.40_(2)_	0.41
PFHpA	11.04_(4)_	2.07	1.57_(2)_	0.16
PFOA	26.18_(4)_	2.11	2.99_(2)_	0.40
PFNA	12.31_(4)_	1.24	<MDL	
PFDA	14.04_(4)_	1.00	<MDL	
PFUnDA	10.46_(4)_	1.03	1.16_(1)_	NA
PFDoA	1.12_(4)_	0.12	0.13_(2)_	0.01
PFTrDA	0.28_(4)_	0.03	<MDL	
PFBS	2.94_(4)_	0.69	1.39_(2)_	0.82
PFPeS*	0.28_(4)_	0.05	0.08_(2)_	0.04
L-PFHxS	1.47_(4)_	0.69	0.73_(2)_	0.10
Br-PFHxS	0.23_(4)_	0.08	0.12_(1)_	NA
PFHpS	2.30_(1)_	NA	<MDL	
L-PFOS	15.04_(4)_	1.79	1.73_(2)_	0.24
Br-PFOS	4.83_(4)_	0.76	0.78_(2)_	0.40
FHxSA	0.26_(4)_	0.06	<MDL	
FHpSA*	0.09_(4)_	0.02	<MDL	
FOSA	0.42_(4)_	0.33	0.15_(1)_	NA
4:2 FTS	0.05_(4)_	0.01	<MDL	
6:2 FTS	1.33_(4)_	0.39	0.63_(1)_	NA
10:2 FTS*	0.16_(4)_	0.02	<MDL	

a Included are the number (*n*) of above-MDL detections that were averaged. Starred compounds (*) are those that have not yet had model-based sampling rates verified in laboratory experiments.

In general, PFAS concentrations were greater at RP1 (former mill) than RP2 (current mill), with ΣPFAS (PFAS detected = 26 for RP1, 16 for RP2) of 120 and 17 ng L^–1^ total PFAS, respectively. Long-chain PFAS were also more prevalent at RP1, likely because emissions at RP1 occurred before initial phase-outs of these compounds after 2000.^31^ Based on this data, RP1 is of greater concern for public and ecological health due to its much higher concentration of regulated PFAS (e.g., PFOA, PFOS).^32^

### PFAS in Sediment

Individual PFAS concentrations in sediment were again higher in RP1 (<MDL—74 ng g^–1^) sediment than RP2 (<MDL—10 ng g^–1^), except for PFBS, which was <MDL at RP1, potentially due to the more recent use of shorter-chain PFAS. In both sediments, long-chain carboxylic acids were the most abundant PFAS (see Table S6). Concentrations of PFAS in sediment for each site were summarized by the maximum detected concentration of each PFAS in any core section (Table 2) and a histogram of depths with maximum concentration (Figure S1).

**Table 2:** Maximum PFAS Concentration Detected in Sediment Core Samples for RP1 and RP2

compound	RP1 (ng g^–1^)	RP2 (ng g^–1^)
PFBA	0.34	0.08
PFPeA	0.78	0.24
PFHxA	1.03	0.23
PFHpA	1.16	0.12
PFOA	3.57	0.60
PFNA	2.58	0.25
PFDA	9.63	2.48
PFUnDA	38.78	6.76
PFDoA	49.63	6.33
PFTrDA	73.64	10.22
PFTeDA	31.01	2.34
PFBS	<MDL	1.01
PFPeS	<MDL	0.03
L-PFHxS	0.08	0.06
PFHpS	<MDL	0.06
L-PFOS	6.82	1.20
Br-PFOS	0.94	0.15
PFDS	0.24	0.18
FHxSA	0.17	<MDL
FHpSA	0.12	<MDL
FOSA	15.85	0.17
6:2 FTS	0.28	<MDL
8:2 FTS	1.23	0.03
10:2 FTS	6.63	0.18

ΣPFAS concentrations in upper layers of RP1 sediment (243 ng g^–1^) were higher than those in AFFF-impacted lakes in Sweden^33^ (76 ng g^–1^), driven partly by a greater number of PFAS detected (*n* = 24 and 6 respectively). Compared to an AFFF-impacted lake in Canada,^34^ higher sum concentrations were found in RP1 for mutually analyzed PFAS (159 vs 16 ng g^–1^ for *n* = 11 mutually analyzed PFAS). Long chain PFCAs (PFOA and larger) drove this difference, with individual concentrations 1–2 orders of magnitude higher at RP1 than the maximum sediment detections by Awad et al. RP1 sediment was similar to urbanized watersheds in Nevada^35^ (ΣPFAS = 273–376 ng g^–1^).

Trends of PFAS concentrations with depth were similar for all PFAS at each site (Figure 2). RP1 displayed PFAS concentrations increasing from the sediment surface to ∼3 cm depth and then decreasing rapidly toward <MDL at 6 cm. RP2 sediment PFAS concentrations were similar from the surface until 3–4 cm depth and then also decreased to negligible quantity deeper than 5 cm. The magnitude of concentrations varied considerably with carbon chain length (e.g., PFTrDA was 20 times more abundant than PFOA). The trend at RP1 likely represented historical emission of high PFAS quantities followed by dormancy where additional sediment accumulated above the peak contamination and/or surface sediment leached PFAS to surface water (acting as the long-term source). Sediment cores from RP2 indicate constant, but low concentration, emission of PFAS over time and more consistent concentrations over both sediment depth and the water column. The decrease in PFAS concentration below 4–5 cm depth at both sites was likely a result of the sediment phase transition to inorganic sand at this depth (for details, see Table S6). As such, PFAS concentrations in sediment qualitatively varied with organic carbon content at RP1, with 5.11–7.40% OC in the top 4 cm of sediment before decreasing to 1.5% beyond 4 cm depth (for details, see Table S6). This aligns with various other studies’ findings for PFAS affinity for organic carbon in sediments.^25,36,37^ The transition to sand at such shallow depth may reduce PFAS sorption quickly beneath the lagoon sediment surface but may result in PFAS transport to deeper groundwater. Thorough hydrological characterization of the site would be necessary to confirm PFAS infiltration in groundwater. Additionally, in future work, deeper sediment coring will be necessary to determine the extent of PFAS contamination and sediment type beyond the upper layers of sediment obtained by hand coring in this study. At RP2, PFAS concentrations did not vary as closely with organic carbon content (Table S6), but this may partially reflect the much lower concentrations present at that site.

**Figure 2:**
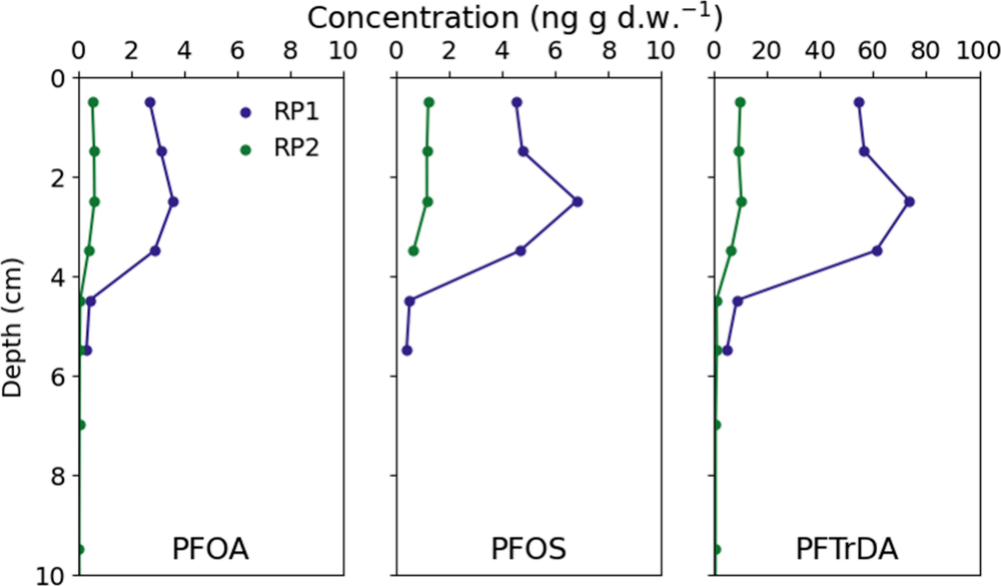
PFAS concentration (ng/g) in sediment over the sediment core depth at each site.

### Laboratory *K*_d_ Determination

Given the potential impact of PFAS releases on water quality and ecological health to the adjacent river, site RP1 was selected for additional investigation of the PFAS’ sediment-water mobility. Log *K*_d_ values were calculated for all PFAS which were detected in both phases in a laboratory batch experiment (for some long-chain PFAS, which were <MDL in the water, a *K*_d_ value was estimated by replacing the missing water concentration with 1/2 MDL, see Figure S2, Table S7). Laboratory-derived log *K*_d_ values ranged from 1 to 5 except for FPeSA. PFAS’ *K*_d_ values generally increased with carbon chain length within functional group classes (log *K*_d_ = 1.6 and 4.7 for PFBA and PFTeDA, respectively). Despite falling in a similar overall range, individual PFAS log *K*_d_ (or corresponding log *K*_OC_ when normalized to organic carbon) in this study differed from previous reports by a log unit or more (both higher^25,33^ and in both directions^38,39^ ) but appeared more consistent with expected trends with carbon chain length (higher sorption for long-chain PFAS). Previous studies indicate that specific sediment mineral content affects partitioning,^25,39^ which was not measured in this study and may account for *K*_d_ and *K*_OC_ differences. The difficulty of detecting some PFAS in the water phase of the *K*_d_ experiment indicated strong sorption/attenuation (sequestration to the solid phase) to sediment, though leaching back to the water column could occur under field conditions. The long-chain PFAS, which were not detectable in the laboratory experiment water phase (PFTrDA and FOSA), would be more likely to leach back in the water column due to sediment resuspension or decreases in OC content (remineralization).

### Comparison with Observed Field *K*_d_

Laboratory-derived *K*_d_ values were compared to those from the field. The latter was estimated from the surficial (0–1 cm) sediment concentration and the overlying water concentrations (Figure 3, Table S7). The apparent field *K*_d_ values from RP1 displayed a good linear correlation with laboratory log *K*_d_ (*R* ^2^ = 0.965, *n* = 16) and slope less than 1 (*p* < 0.0001), indicating disequilibrium between sediment and water in the field. In general, apparent field *K*_d_ values deviated more from equilibrium with increasing *K*_d_, i.e., long-chain PFAS, which partition strongly to sediment, were below dissolved concentrations expected from equilibrium with the sediment, which might reflect a combination of dilution, flushing, or water column stripping to colloidal or particulate phases. FPeSA did not agree with the overall trend and potentially indicated water-phase degradation of these compounds in the field. Some PFAS have shown biodegradability in the environment, but the mechanisms and rates are unknown.^40–42^ Overall, PFAS at the site were depleted in the water column.

**Figure 3:**
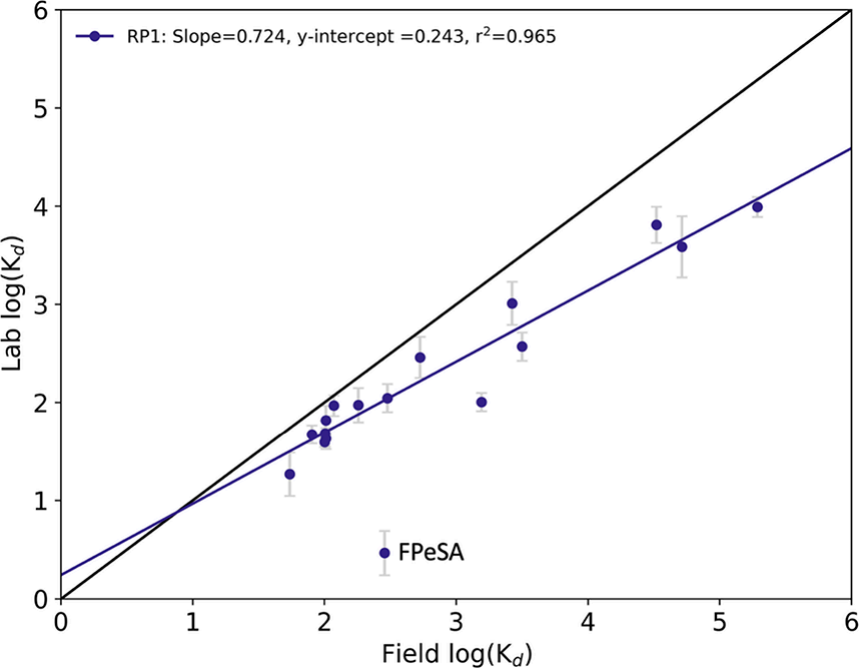
Comparison of laboratory determined log *K*_d_ values and observed field log *K*_d_ for RP1. FPeSA was excluded from linear regression analysis.

### PFAS Diffusion out of the Sediment

The effective diffusion distance of the PFAS out of the contaminated sediment layers varied from <1 mm per year for PFTeDA (see Table 3) to over 6 mm per year for FPeSA. A net sedimentation rate of ∼1 mm year^–1^ can be derived for the sediment core, where a majority of PFAS were contained in the top 6 cm depth of PFAS contamination, coupled with an approximately 60 year pollution history of the waste lagoons (ca. 1965).^26^ Comparing the net sediment accumulation rate with the estimated diffusion distances enables an approximation of which sedimentary PFAS can be considered mobile and for which the sediment represents a long-term storage (final sink). A size-based cut off emerged at PFTrDA. PFAS up to PFTrDA could easily diffuse out of sediment, faster than the sedimentation rate vs PFTeDA, which diffuses too slowly and partitions too strongly to do so when sediment is undisturbed. Various sources of uncertainty exist in this estimation, especially the variability of these values over the large pond surface area. However, the sensitivity analysis for model results indicated that a 30% change in any variable affects the diffusion travel distance model by less than 20% (Table S4). The estimation of travel distance and PFAS, which cannot travel faster than sedimentation, is robust to input uncertainty, and the included propagated uncertainties are most useful for evaluating these model results.

**Table 3:** Estimated Effective Diffusion Travel Distance for Each PFAS in RP1 Sediment^a^

compound	*X*_1/2_ (cm year^–1^)	St. Dev.
PFBA	2.08	0.42
PFPeA	1.78	0.68
PFHxA	1.23	0.37
PFHpA	1.65	0.43
PFOA	1.35	0.57
PFNA	1.10	0.55
PFDA	0.61	0.38
PFUdA	0.32	0.21
PFDoA	0.12	0.07
PFTrDA	0.10	0.03
PFTeDA	0.04	0.04
L-PFHxS	2.54	1.70
PFHpS	1.80	0.17
L-PFOS	0.99	0.40
Br-PFOS	1.59	0.46
FPeSA	5.99	4.13
FOSA	0.11	0.05
6:2FTS	1.08	0.27
8:2FTS	0.53	0.21
10:2FTS	0.16	0.17

a Uncertainty was calculated from the measured *K*_d_ and calculated *D*_w_.

### Desorption Time-Scales from the Sediment

For the PFAS (up to PFTrDA), which are likely mobile enough to diffuse out of the sediment, sedimentary loss fluxes were calculated in a simple 2-box model (for details, see Methods). Loss fluxes from the sediment varied between 5 and 228 μg m^–2^ year^–1^ (Table 4). Assuming a constant mass flux out, we derived the minimum time needed for desorption of the total mass bound in the sediment for each PFAS. Minimum desorption times ranged from three years for FPeSA to >100 years for PFDoA, PFTrDA, and FOSA (Table 1).

**Table 4:** Estimated Flux of Mobile PFAS from RP1 Sediment (μg m^–2^ year^–1^) and Minimum Sediment Depletion Time (years) for Desorbing PFAS, Assuming a Constant Flux^a^

compound	flux (μg m^–2^ year^–1^)	desorption time (years)
PFBA	14.3 ± 3.5	7
PFPeA	22.6 ± 12.1	9
PFHxA	23.3 ± 10.1	13
PFHpA	35.1 ± 14.1	9
PFOA	85.3 ± 50.2	12
PFNA	60.2 ± 43.0	13
PFDA	111.5 ± 98.2	24
PFUdA	227.5 ± 212.6	49
PFDoA	126.5 ± 95.8	108
PFTrDA	156.0 ± 61.0	131
L-PFHxS	5.4 ± 5.7	5
L-PFOS	117.2 ± 67.5	15
Br-PFOS	20.2 ± 8.7	11
FPeSA	5.1 ± 5.0	3
FOSA	23.1 ± 17.3	189
6:2 FTS	8.0 ± 3.0	11
8:2 FTS	18.7 ± 12.2	21
10:2 FTS	28.0 ± 43.2	67

a PFAS, which did not have mobility distances greater than 0.1 cm (including uncertainties), were excluded, as well as PFHpS as it was <MDL in 0–1 cm sediment. Uncertainty values in flux amounts were propagated from measured water and sediment concentrations, calculated *D*_w_, and measured *K*_d_ values.

Under the current conditions, PFUdA has the highest mass flux (228 μg m^–2^ year^–1^) out of RP1 sediment due to its relative mobility and high disparity between field distributions and equilibrium. If current flux rates remained constant, it would require about 50 years of constant sediment desorption and water flushing to remove all PFUdA from the 6 cm contaminated sediment naturally. These conditions are unlikely at the steady state in RP1, though, as rainfall, additional PFAS inputs, sediment resuspension, bioturbation, and other natural processes will affect mobility and equilibrium conditions.^30,43^ Rather, these simplified estimations demonstrate (1) the ability of various PFAS to diffuse out of sediment faster than sedimentation and (2) the potential diffusion-based fluxes from sediment under the specific conditions during field sampling. Despite various sources of uncertainty and certainty of other processes, the calculations performed here show the potential for RP1 to constitute a source of sedimentary PFAS contamination over decades. This input of PFAS to the Pawcatuck River is especially notable given the river’s few other direct sources of PFAS.

Based on the sensitivity analysis, flux model calculations were most influenced by changes in measured sediment porosity (up to a factor of ∼2 change in flux from 30% porosity variation). This is a result of its repeated use in the flux model calculations. Higher porosity decreases estimated flux due to the lowered estimation of PFAS stored in the sediment/porewater compartment (i.e., greater porosity = more porewater volume, which contains less PFAS). Thus, the concentration gradient and therefore flux between a more porous sediment/water mixture and open water are smaller relative to more compact sediment. While porosity was not included in error propagation (it was a single point measurement in the laboratory), our measurement is consistent with other freshwater pond upper sediment layers.^44,45^ As expected, the measured sediment concentrations were the second most important variable affecting flux calculations, but the impact of varying by ±30% was less than half as changes in porosity. Thus, sediment porosity was identified as the most important parameter to the overall uncertainty in flux calculations above.

## Conclusions

Site RP1 represents a source of long-term contamination and contribution of PFAS to the Pawcatuck River in Rhode Island. PFAS are present in high quantities in both sediment and water at RP1 (up to 74 ng g^–1^ PFTrDA and 26 ng L^–1^ PFOA), and *K*_d_ values indicate a range from high mobility to long-term storage. Several PFAS of great concern for public health (such as PFOA, PFNA, PFDA, PFOS, 6:2 FTS, and 8:2 FTS) were estimated to require decades to be fully removed from sediment at current flux rates at RP1, with others potentially requiring over 100 years to be removed. However, this natural removal simply constitutes a redistribution to other environmental matrices, such as the water column and biota. At RP1, the upper centimeters of sediment would require remediation to address the potential long-term PFAS source there (RP2’s PFAS contamination is of lesser concern). Without remediation, notable concentrations of PFAS such as those measured in Dunn et al.^27^ in the Pawcatuck River will continue downstream of RP1. Various treatment technologies are under development for the remediation of sediment and other environmental matrices (usually *ex situ*),^46^ but with few full-size demonstrations. Locations such as RP1 in Rhode Island may be an ideal site for field scale demonstration of emerging sediment remediation technologies due to the shallow sediment contamination and accessible and contained locations of the retention ponds.

## Supplementary Material





## Notes

Additional details on sampling sites, LC-MS/MS analysis, passive sampling, and transport models (PDF)
LC-MS/MS target compound information, QA/QC values, and numerical experimental results (XLSX)
