# Supplementary material for: Characterization of the Potential Long-Term Impact from Sedimentary PFAS at a Historically Contaminated Textile Waste Site
Source: ACS ES T Water. 2025 Dec 29;6(1):521–8. doi: 10.1021/acsestwater.5c01210 (PMC12797218; doi:10.1021/acsestwater.5c01210)
Supplement: Supplementary file 1 [file ew5c01210_si_001.pdf]

Supplemental Information

Characterization of Potential Long-Term Impact from Sedimentary PFAS at Historically Contaminated Textile Waste Site

Jarod Snook[1], Jitka Becanova[1], Simon Vojta[1], and Rainer Lohmann[1*]

[1]Univerisity of Rhode Island Graduate School of Oceanography. 215 S Ferry Rd, Narragansett, RI, 02882, United States.

*corresponding author, Rainer Lohmann (rlohmann@uri.edu)

Table of Contents

Supplemental Map. Sampling Sites	……………………………………………S3
Supplemental Text 1. Sampling Site Details	……………………………………………S3
Supplemental Text 2. Extraction and K_d Experiment Details	……………………………………………S4
Table S1. Sampling Rates for DGT Passive Sampling	……………………………………See Excel
Supplemental Text 3. LC-MS/MS analysis details.	……………………………………………S5
Table S2. Target Compound List for LC-MS/MS Analysis	……………………………………See Excel
Table S3. Method Detection Limits for Various Sample Types	……………………………………See Excel
Table S4. Internal Standard Recoveries for DGT Passive Samplers	……………………………………See Excel
Table S5. Sensitivity Analysis Results	……………………………………See Excel
Table S6. Sediment Core PFAS Concentrations, Core Description, and Organic Carbon Content	…………………………………… See Excel

Figure S1. Histogram of sediment core depths where PFAS maximum concentrations were detected at RP2 ……………………………………………S5

Figure S2. Laboratory-measured K_d Values for RP1 ……………………………………………S6

Table S7. K_d Values Calculated in Laboratory Batch Experiment and Field Sampling …………………………………….See Excel

Supplemental Map. Retention Pond field sites near Westerly, Rhode Island. Interconnected pong pathways are highlighted in orange, and the Pawcatuck River is traced in blue. Green stars are specific sampling sites for each retention pond.

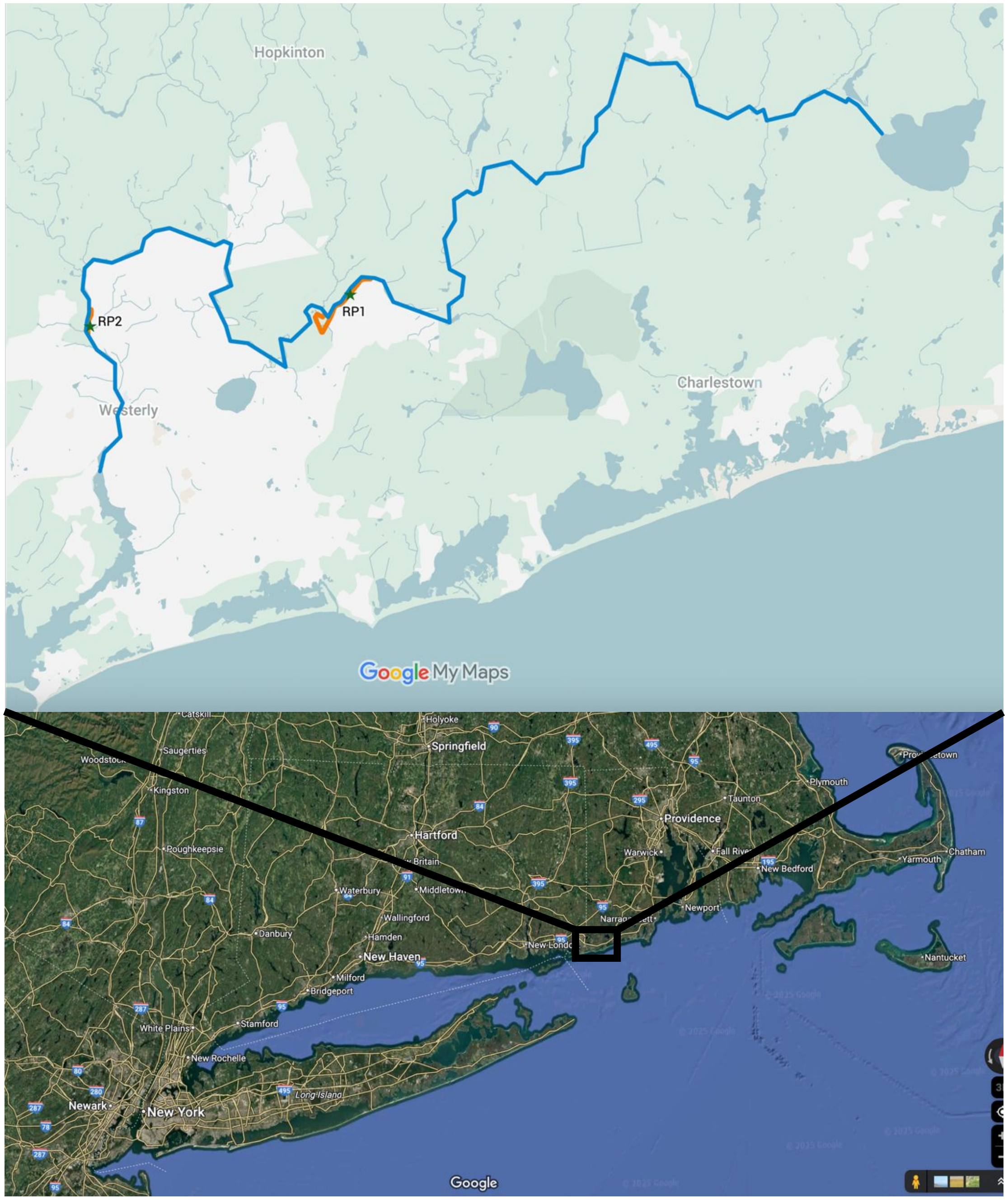

Supplemental Text 1. Sampling Site Details

Retention Pond 1 (RP1) is in Bradford, Rhode Island (coordinates: 41.4035, -71.7593) and is one in a series of man-made and natural freshwater ponds parallel to the Pawcatuck River. Between the 1960s—1980s, the interconnected ponds served as settling lagoons for waste products produced at a nearby, now defunct textile mill[1], travelling for approximately 2.7 km before feeding into the river. At the chosen site, water depth was 2 m. Water temperature in the pond rose from 11.4—23.5°C over the sampling campaign, with a time-weighted average of 16.7°C. Six DGT samplers were deployed at RP1.

Retention Pond 2 (RP2) in Westerly, Rhode Island (coordinates: 41.3957, -71.8419) is a man-made freshwater retention pond/canal running for 600 m parallel to the Pawcatuck River before flowing into the river. Per previous research[2], the pond has a separate PFAS signature, potentially due to the currently active nature of the nearby textile mill. The water depth at RP2 was 1.5 m. The water temperature similarly rose over the sampling campaign, from 12.0—20.5°C (average: 14.2°C). Six DGT passive samplers were deployed at RP2. One moderate rainfall occurred (one 1.2 cm 24-hour rain event May 6) over deployment period April 23—May 7 affecting both sites. Due to the nature of passive sampler measured water concentrations, the results are minimally affected by this rain event.

Supplemental Text 2. Extraction Details for DGT, and sediment, and K_d experiment details

DGT

Upon recovery, the general extraction procedure is as follows: The PFAS-retaining layer is isolated, spiked with PFAS internal standard, and freeze dried. It is then extracted twice with 1% ammonium hydroxide in methanol solution on a shaker table. Extracts are concentrated by evaporation and analyzed with LC-MS/MS. Sampling rates, determined and validated previously, were used to convert the accumulated PFAS mass on the sampler to a dissolved water concentration, adjusting for temperature if necessary. Other water properties do not affect the sampling rate of DGT.[3] In this case, sampling rates were adjusted for the time-weighted average temperature observed over the sampling period at each site (Table S1).

Sediment

Sediment subsamples were isolated and spiked with internal standard for PFAS analysis. Each sediment sample was extracted once with 5 mL LC-MS grade methanol for 24 hours, then centrifuged, methanol poured off and extracted again with 5 mL 1% ammonium hydroxide in methanol solution. The combined extracts were concentrated via nitrogen and dry bath evaporator, cleaned with Envi-Carb solid-phase extraction cartridges, and analyzed with LC-MS/MS. As PFAS-free sediment was not available for blanks, methanol-solution process blanks were used to calculate method detection limits.

K_d Experiment Details

Sediment extraction followed the same procedure described above, and water extraction utilized a modified version of EPA Method 1633 (solid-phase extraction with weak-anion exchange adsorbent cartridge).[4,5] Final water concentrations were higher, and sediment in general the same order of magnitude, as field-measured values indicating no phase depletion issues in the laboratory K_d experiment (though some expected PFAS concentrations in water were below MDL due to the relatively small sample quantity used).

Supplemental Text 3. LC-MS/MS analysis

The instrumental analysis was performed using a SCIEX ExionLC AC UHPLC system coupled to a SCIEX X500R quadrupole time-of-flight tandem mass spectrometer (QTOF MSMS). A Phenomenex Gemini 3um C18 110Å 50x2mm LC analytical column preceded with a Phenomenex SecurityGuard cartridge was used for the analyte separation. Another Phenomenex Gemini 5um C18 110 Å 50x4.6mm LC analytical column was used to delay the PFAS instrumental contribution. The aqueous mobile phase (MPA) was 10mM ammonium acetate in water, and the organic mobile phase (MPB) was 10mM ammonium acetate in methanol. LC parameters were set to: flow 0.3mL/min, injection 20uL, column oven 45°C. Solvent gradient of MPB gradually increased from 40% to 80% (1 to 5.5 min), 80% to 100% (5.5 to 7 min), then hold for one minute and finally drop to 40% (8 to 8.5 min) and hold for another 6.5 minutes. For the quantification of the target analytes, a HRMSMS (MRM HR) method was used. Negative mode ESI with the following parameters was used: curtain Gas at 30 psi, ion source gas 1 at 40 psi, ion source gas 2 at 60 psi, temperature 450°C.

Figure S2. Histogram of sediment core depths where PFAS maximum concentrations were detected at RP2.

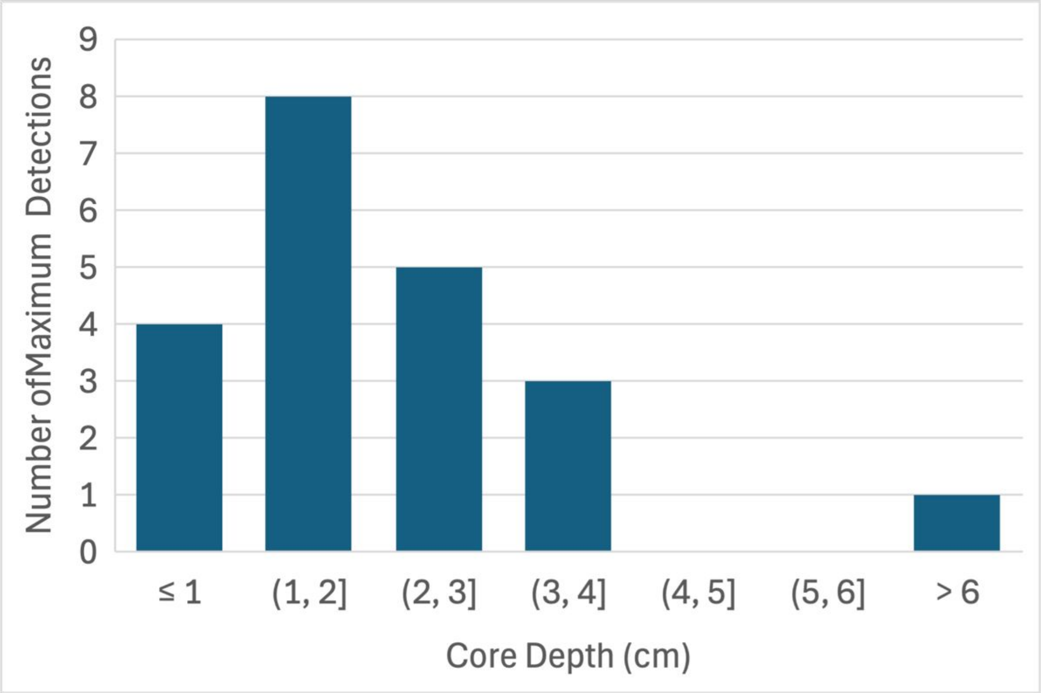

Figure S3. Laboratory experiment log(K_d) values. Error bars are the (log) standard deviation of replicates, and starred bars were estimated with water concentration replacement with ½ MDL. Bars lacking both error bars and asterisks (PFHpS) had only one replicate with successful K_d determination.

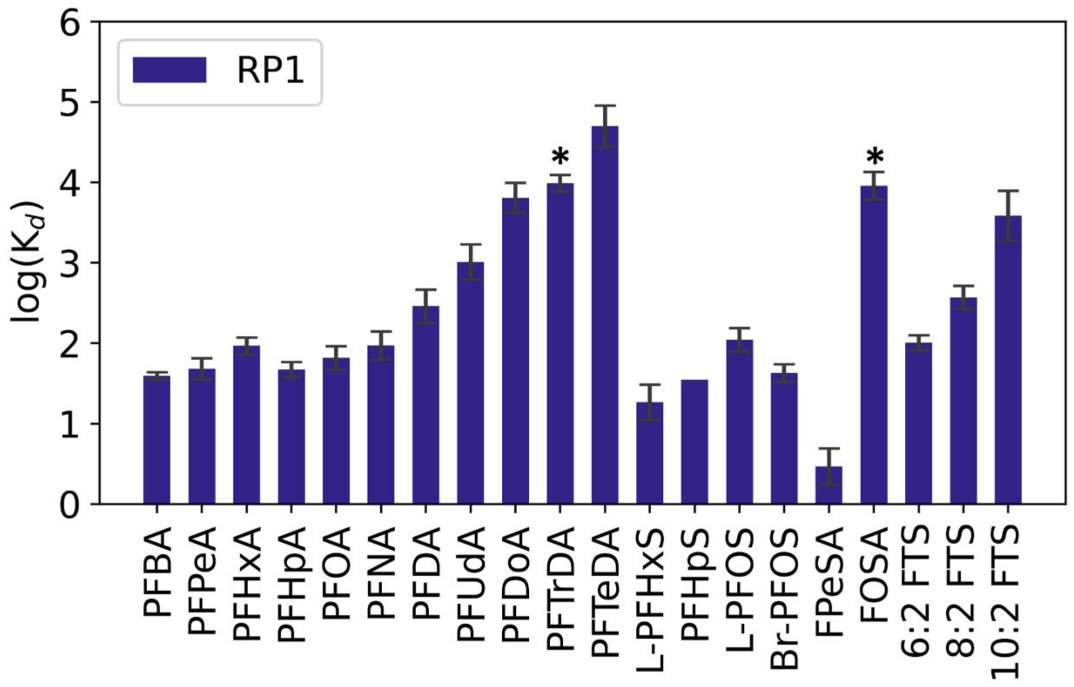

References

(1) Rhode Island Department of Health. Health Consultation - BRADFORD DYEING ASSOCIATION: PFAS IN FISH TISSUE. **2024**.

(2) Dunn, M.; Noons, N.; Vojta, S.; Becanova, J.; Pickard, H.; Sunderland, E. M.; Lohmann, R. Unregulated Active and Closed Textile Mills Represent a Significant Vector of PFAS Contamination into Coastal Rivers. *ACS EST Water* **2024**, *4* (1), 114–124. https://doi.org/10.1021/acsestwater.3c00439.

(3) Snook, J.; Becanova, J.; Vojta, S.; Lohmann, R. Furthering the Capabilities of Diffusive-Gradient Passive Samplers for Per- and Polyfluoroalkyl Substances. *Environ. Sci. Technol.* **2025**, *59* (19), 9744–9753. https://doi.org/10.1021/acs.est.4c14136.

(4) Becanova, J.; Saleeba, Z. S. S. L.; Stone, A.; Robuck, A. R.; Hurt, R. H.; Lohmann, R. A Graphene-Based Hydrogel Monolith with Tailored Surface Chemistry for PFAS Passive Sampling. *Environ. Sci. Nano* **2021**, *8* (10), 2894–2907. https://doi.org/10.1039/D1EN00517K.

(5) EPA. 3rd Draft Method 1633 Analysis of Per- and Polyfluoroalkyl Substances (PFAS) in Aqueous, Solid, Biosolids, and Tissue Samples by LC-MS/MS. **2022**.
